# Pediatric orbital IgG4-related disease: A case series

**DOI:** 10.1016/j.ajoc.2026.102612

**Published:** 2026-06-06

**Authors:** Hassan Asadigandomani, Afsaneh Malekpour, Mohammad Taher Rajabi, Amirhossein Aghajani, Fahimeh Asadi Amoli, Seyed Mohsen Rafizadeh

**Affiliations:** a Department of Ophthalmology, University of California San Francisco, San Francisco, CA, 94143-4081, USA; b Department of Oculofacial Plastic and Reconstructive Surgery, Farabi Eye Hospital, Tehran University of Medical Sciences, Tehran, Iran; c Department of Pathology, Farabi Eye Hospital, Tehran University of Medical Sciences, Tehran, Iran

**Keywords:** IgG4-related disease, Immunosuppressive therapy, Lacrimal gland, Orbit, Pediatrics, Proptosis

## Abstract

**Purpose:**

Immunoglobulin G4 (IgG4)-related orbital disease is an uncommon fibroinflammatory condition characterized by IgG4-positive plasma cell infiltration of orbital structures. While its features are well-recognized in adults, pediatric cases remain exceedingly rare, with limited reports in the literature.

**Observations:**

We describe three pediatric female patients with IgG4-related orbital disease, aged 5, 11, and 16 years, who presented with progressive proptosis and globe displacement. Orbital imaging in all cases demonstrated infiltrative lesions involving the lacrimal gland. Histopathologic confirmation was obtained in each, showing dense lymphoplasmacytic infiltrates with cluster of differentiation 138 (CD138) positivity and a high proportion of IgG4-positive plasma cells. Treatment primarily consisted of systemic corticosteroids and immunomodulatory therapy. Rituximab was successfully administered in one refractory case, leading to substantial clinical improvement.

**Conclusions and importance:**

Pediatric IgG4-related orbital disease, although rare, should be considered in the differential diagnosis of orbital masses in children, particularly when the lacrimal gland is involved. Histopathology remains essential for diagnosis, and systemic corticosteroids constitute the mainstay of therapy, with immunosuppressants and biologics reserved for refractory disease.

## 1 Introduction

Immunoglobulin (Ig) G4-related orbital disease represents a subset of IgG4-related disease characterized by chronic fibroinflammatory infiltration of the orbit, most commonly affecting the lacrimal glands, extraocular muscles, and branches of the trigeminal nerve.^1^ The condition can manifest clinically as proptosis, eyelid swelling, diplopia, or conjunctival inflammation.^1^ Although the median age of onset in adults is around 60 years, pediatric presentations remain extremely uncommon.^2^

Diagnosis relies on a combination of clinical, radiological, and histopathological findings. Imaging typically demonstrates enlargement of the lacrimal gland or extraocular muscles, while histology reveals dense lymphoplasmacytic infiltration with storiform fibrosis, obliterative phlebitis, and an increased IgG4/IgG plasma cell ratio (≥40%) or more than 40 IgG4-positive cells per high-power field (HPF). Elevated serum IgG4 levels (>135 mg/dL) may support the diagnosis but are not essential.^3^

First-line therapy consists of systemic corticosteroids and disease-modifying antirheumatic drugs (DMARDs), including methotrexate, mycophenolate mofetil, and azathioprine. Refractory cases may benefit from biologic therapy such as rituximab or infliximab.^4^

Orbital involvement was found in less than half of pediatric patients with IgG4-related disease, with cases ranging from 5 to 15 years of age.^5^, ^6^, ^7^, ^8^, ^9^ Therefore, its clinical presentation and response to therapy warrant further investigation. Herein, we present three cases of pediatric IgG4-related orbital disease to highlight their clinical spectrum, diagnostic features, and therapeutic outcomes. All patients underwent clinical, radiologic, and histopathologic evaluation, with no evidence of systemic vasculitis, necrotizing granulomatous inflammation, lymphoproliferative disease, lymphoma, or infectious etiology.

### 1.1 Case 1

A 5-year-old girl presented with several months of progressive bilateral proptosis and eyelid swelling. There was no history of trauma or systemic illness. Best-corrected visual acuity (BCVA) was 20/32 in the right eye and 20/200 in the left eye. Examination revealed bilateral eyelid erythema, chemosis, and 5 mm of proptosis on Hertel exophthalmometry, with moderate restriction of ocular motility in all gazes. Pupillary reactions were normal in both eyes, and fundus examinations were unremarkable.

Orbital computed tomography (CT) scan without contrast revealed bilateral infiltrative orbital soft tissue masses involving both lacrimal glands, with involvement of all extraocular muscles and their tendons, as well as enlargement of the left infraorbital nerve. The lesions demonstrated posterior extension toward the orbital apex with molding of the globes. No calcification or bone destruction was noted. Contrast-enhanced magnetic resonance imaging (MRI) demonstrated bilateral homogeneously enhancing superolateral orbital lesions with irregular margins.

The patient underwent a left superolateral orbitotomy with tumor biopsy. Histopathologic analysis showed dense lymphoplasmacytic infiltration with cluster of differentiation 138 (CD138) positivity and approximately 25 IgG4-positive plasma cells per HPF, confirming IgG4-related disease. Serum IgG4 level was within normal limits. Systemic review and multidisciplinary evaluation revealed no evidence of extraorbital IgG4-related disease or other systemic involvement.

**Fig. 1:**
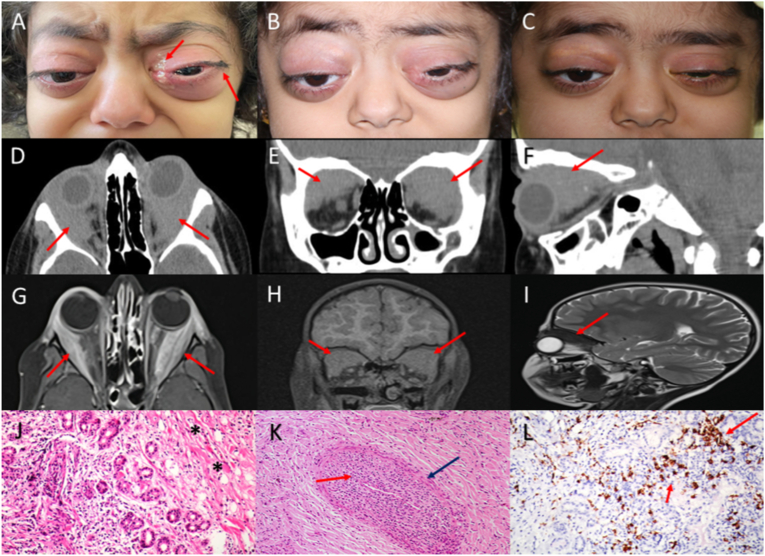
Clinical, radiologic, and histopathologic findings of a 5-year-old girl with bilateral immunoglobulin G4 (IgG4)-related orbital disease. (**A**) Pretreatment clinical photograph showing bilateral proptosis, eyelid swelling, and conjunctival chemosis, with medial and lateral tarsorrhaphy performed in the left eye (arrows) for corneal protection against exposure keratopathy. (**B**) Clinical improvement 3 months after treatment with rituximab. (**C**) Further improvement at 1-year follow-up after treatment. (**D-F**) Orbital computed tomography (CT) scans: axial (**D**), coronal (**E**), and sagittal (**F**) views demonstrating bilateral extraconal infiltrative homogeneous masses with irregular borders (arrows), molding the superolateral orbital walls and displacing the globes inferiorly. (**G-H**) Contrast-enhanced orbital magnetic resonance imaging (MRI) demonstrating bilateral homogeneously enhancing lesions (red arrows) on T1-weighted images, located superolateral to the globes, with irregular margins. (**I**) Sagittal T2-weighted MRI showing the extent of the orbital lesion (red arrow). (**J**) Histopathology showing dense lymphoplasmacytic infiltration and fibrosis (asterisks) on hematoxylin and eosin (H&E) staining. (**K**) Obliterative phlebitis (blue arrow); the vein wall is preserved, while the lumen is nearly completely replaced by inflammatory infiltrate (red arrow) on H&E staining. (**L**) Immunohistochemical (IHC) staining of lacrimal gland tissue demonstrating cluster of differentiation 138 (CD138)-positive plasma cells (red arrows) with increased numbers of IgG4-positive plasma cells. (For interpretation of the references to colour in this figure legend, the reader is referred to the Web version of this article.)

The patient received systemic corticosteroids and multiple immunosuppressive agents, including methotrexate, azathioprine, cyclosporine, and mycophenolate mofetil, but her disease remained refractory. Due to progressive proptosis and exposure keratopathy in her left eye, lateral and medial tarsorrhaphy were performed for corneal protection. Rituximab therapy was then initiated, resulting in marked bilateral reduction of proptosis, chemosis, and eyelid swelling, with resolution of left eye exposure keratopathy within three months. The patient was followed for 1 year, with sustained clinical improvement and no evidence of recurrence (Fig. 1).

### 1.2 Case 2

An 11-year-old girl presented with a one-month history of progressive right eye proptosis without pain or diplopia. There was no positive history of trauma or systemic disease. BCVA was 20/20 in both eyes. On examination, mild right upper eyelid ptosis and 2 mm of right-sided proptosis were noted on Hertel exophthalmometry. Relative afferent pupillary defect (RAPD) was absent. There was no chemosis, eyelid swelling, or limitation of eye movements, and fundus examination was normal bilaterally.

**Fig. 2:**
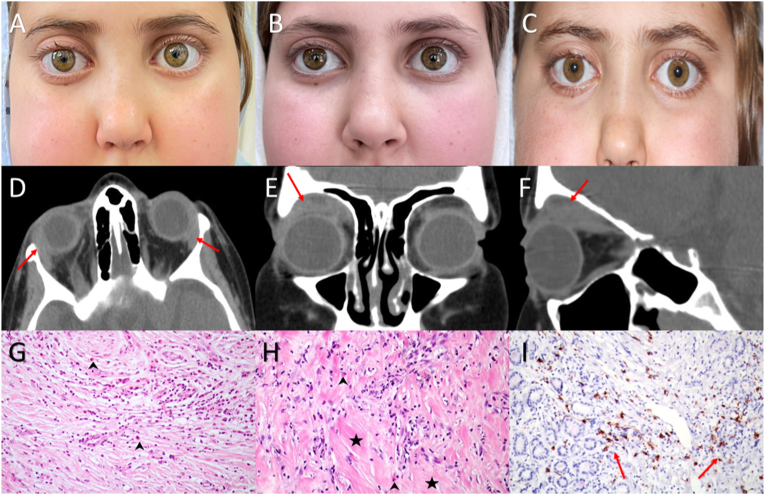
Clinical, radiologic, and histopathologic findings of an 11-year-old girl with bilateral immunoglobulin G4 (IgG4)-related lacrimal gland disease, more prominent on the right side. (**A**) Pretreatment clinical photograph showing mild right upper eyelid ptosis and right-sided proptosis. **(B)** Clinical improvement 3 months after treatment. **(C)** Further improvement at 1-year follow-up after treatment. **(D**–**F)** Orbital computed tomography (CT) scans: axial (**D**), coronal (**E**), and sagittal (**F**) views showing homogeneous bilateral lacrimal gland masses with irregular borders, without bone invasion or intraconal extension (arrows). **(G)** Histopathology showing mixed chronic inflammatory cell infiltration, predominantly composed of lymphoplasmacytic cells admixed with eosinophils (arrowheads), within a fibrotic stroma on hematoxylin and eosin (H&E) staining. **(H)** Storiform fibrosis with inflammatory cell infiltration, predominantly composed of lymphoplasmacytic cells and eosinophils (arrowheads), with fine collagen fibers (asterisks) arranged in a flowing pattern on H&E staining. **(I)** Immunohistochemical (IHC) staining demonstrating increased IgG4-positive plasma cells and cluster of differentiation 138 (CD138)-positive plasma cells (arrows).

Orbital CT scan without contrast showed bilateral lacrimal gland enlargement, more prominent on the right side, with associated involvement of the right superior rectus muscle, levator palpebrae superioris complex, and their tendons, together with a superior infiltrative orbital mass. There was no bone invasion or intraconal extension. The patient underwent right superior orbitotomy with lacrimal gland biopsy. Histopathology confirmed IgG4-related disease, with CD138 positivity and >25 IgG4-positive plasma cells per HPF. Serum IgG4 level was elevated at 156.24 mg/dL (reference range 6.2–112.7 mg/dL). Further systemic assessment and specialty consultations showed no signs of multisystem IgG4-related disease. She received systemic corticosteroids and azathioprine. At three months, the patient demonstrated significant improvement in right eye proptosis. At 1-year follow-up, the patient remained clinically stable with no evidence of recurrence (Fig. 2).

### 1.3 Case 3

A 16-year-old girl presented with proptosis and globe displacement in the left eye. She had no history of systemic disease or trauma. BCVA was 20/20 in both eyes. Examination revealed left eye proptosis with inferior displacement of the globe. Hertel exophthalmometry measured 5 mm of left-sided proptosis. Ocular motility of the left eye was restricted in abduction and adduction. Pupillary responses were normal, and fundus examinations were unremarkable bilaterally.

A prior incisional biopsy performed at another center had been reported as angiolymphoid hyperplasia, without immunohistochemical (IHC) staining. Orbital CT scan without contrast revealed a diffuse infiltrative mass in the superomedial compartment of the left orbit, extending around the globe and causing globe indentation, with involvement of the anterior optic nerve, superior rectus muscle, levator palpebrae superioris complex, medial rectus muscle, lateral rectus muscle, superior oblique muscle, and lacrimal gland.

**Fig. 3:**
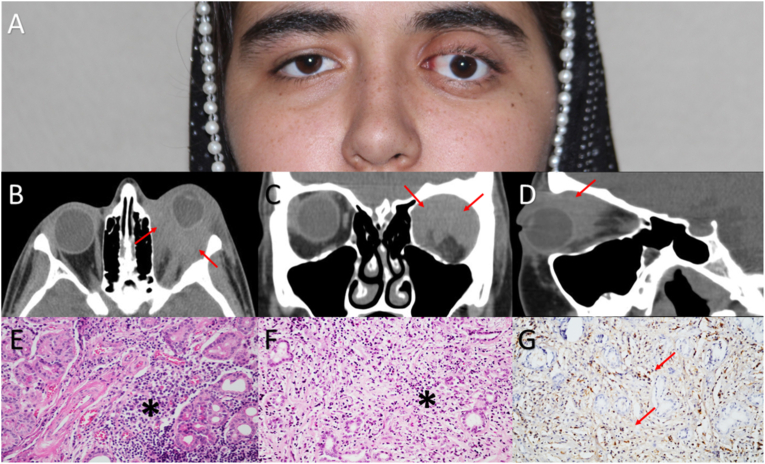
Clinical, radiologic, and histopathologic findings of a 16-year-old girl with left-sided immunoglobulin G4 (IgG4)-related orbital disease. **(A)** Clinical photograph at presentation demonstrating left-sided proptosis and inferotemporal globe displacement. **(B**–**D)** Orbital computed tomography (CT) scans: axial (**B**), coronal (**C**), and sagittal (**D**) views revealing a diffuse, infiltrative, homogeneous mass involving both the intraconal and extraconal spaces, with temporal and inferior displacement of the left globe (arrows). **(E, F)** Histopathologic sections of lacrimal gland tissue showing marked lymphoplasmacytic and eosinophilic infiltration with fibrosis (asterisks) on hematoxylin and eosin (H&E) staining. **(G)** Immunohistochemical (IHC) staining of lacrimal gland tissue demonstrating cluster of differentiation 138 (CD138)-positive plasma cells and increased IgG4-positive plasma cells (arrows).

The patient underwent deep left lateral orbitotomy with excisional biopsy. IHC staining supported the diagnosis of IgG4-related disease, showing CD138 positivity and >40% IgG4-positive plasma cells. Serum IgG4 level was within normal limits. Comprehensive systemic evaluation demonstrated no associated systemic manifestations of IgG4-related disease. She was treated with systemic corticosteroids and mycophenolate mofetil. The patient was followed for 1 year, with gradual regression of proptosis and no evidence of recurrence (Fig. 3).

## 2 Discussion

In this study, IgG4-related disease was identified in three pediatric patients, aged 5, 11, and 16 years, all of whom were female. Progressive proptosis and globe displacement were the primary presenting features in all three cases, with reduced vision noted in one patient. Imaging revealed a homogeneous, infiltrative extraconal mass with irregular borders involving the lacrimal gland in each case. All patients underwent orbitotomy with biopsy, and the histopathologic findings, together with CD138 and IgG4 immunostaining, supported the diagnosis of IgG4-related disease.

IgG4-related disease is classically described in adults, while orbital involvement in the pediatric population remains less well established. Hara et al. reviewed 135 cases of pediatric IgG4-related disease and reported orbital involvement in 40 percent of the cases, suggesting it as a common localized manifestation of disease in children rather than systemic involvement.^10^ They also reported that only 20–27% of published cases met the formal American College of Rheumatology (ACR)/European League Against Rheumatism (EULAR) classification criteria, highlighting a significant diagnostic gap in pediatric IgG4-related disease.^11^ In a systematic review by Karim et al., 25 pediatric cases of IgG4-related disease were identified, with a median age of 13 years and 64% being female.^12^ Orbital manifestations were present in 44% of cases, while other organ involvement included IgG4-related pancreatitis/autoimmune pancreatitis type 1 (12%), IgG4-related cholangitis (8%), and IgG4-related pulmonary disease (8%).^12^ As in our series, diagnosis in nearly all cases was supported by histopathologic findings, with elevated serum IgG4 levels documented in 16/23 (70%) patients. Prednisolone was the first-line treatment in 23/25 cases; 10 patients achieved complete remission with steroids alone, while 11 required additional DMARDs, most commonly mycophenolate mofetil (5 cases), followed by azathioprine and methotrexate. In refractory cases, rituximab, adalimumab, and cyclophosphamide were initiated with reported success.^12^

Similarly, Smerla et al. described a 4-year-old boy with right-sided IgG4-related orbital disease involving the lateral and superior rectus and superior oblique muscles, as well as the lacrimal gland.^5^ Diagnosis was supported histologically from an orbital biopsy, which showed a lymphoplasmacytic infiltrate with storiform fibrosis and more than 10 IgG4-positive plasma cells/HPF, as well as an elevated serum IgG4 level (222 mg/dL). The patient was successfully treated with oral prednisolone administered for two months, with complete radiologic resolution documented at four-month follow-up. Another case report of a girl as young as 4 years old with involvement of the left orbital soft tissues, myositis of the left lateral rectus, and left upper eyelid was reported by Ulas et al., diagnosed based on clinical and radiological findings and marked response to intravenous corticosteroids.^13^

Jalaj et al. presented a 9-year-old female with right lacrimal gland enlargement and symptoms of swelling, proptosis, and tenderness of the right upper eyelid.^14^ The diagnosis of IgG4-related orbital disease was supported histopathologically, meeting the criteria. The initial use of corticosteroids was not successful, but the disease was responsive to adalimumab, establishing it as a viable second-line treatment. Other recent case reports with similar findings in the pediatric population are summarized in Table 1.

**Table 1:** Summary of reported pediatric immunoglobulin G4 (IgG4)-related orbital disease cases.

Article (author, year)	Age	Gender	Laterality	Site of involvement	Diagnostic findings	Treatment and outcome
Ulas et al., 2023^13^	4	Female	Unilateral (left)	Left orbital soft tissue, lateral rectus muscle (myositis), left upper eyelid	Biopsy: chronic inflammation (immunoglobulin G4 (IgG4) stain not specific), serum IgG4 elevated	Rapid response to intravenous and oral steroids in three months
Smerla et al., 2018^5^	4	Male	Unilateral (right)	Right superolateral extraconal soft tissue, lacrimal gland, lateral rectus muscle, superior rectus muscle, superior oblique muscle	Serum IgG4 elevated, Biopsy: lymphoplasmacytic infiltrate, >10 IgG4 positive cells/high-power field (HPF), IgG4/IgG >0.5	Responded to oral prednisolone administered for 2 months, with complete radiologic resolution documented at 4 months.
Jalaj et al., 2018^14^	9	Female	Unilateral (right)	Right lacrimal gland, right superior orbital soft tissue	Biopsy: >100 IgG4 positive cells/HPF, IgG4/IgG >0.4, phlebitis, dense lymphoplasmacytic infiltration	Steroid-refractory, treated with adalimumab (first reported pediatric use)
Meel et al., 2022^7^	12 at first presentation (reported at age 24)	Male	Bilateral	Bilateral intraconal and extraconal orbital masses with extraocular muscle, orbital apex, and optic nerve involvement	Serum IgG4 normal, Biopsy: chronic sclerosing plasma cell-rich inflammation; >25 IgG4 positive cells/HPF	Oral corticosteroids resulted in rapid clinical and radiologic improvement, with stable vision and no recurrence during 4-year follow-up
Bu et al., 2022^9^ (Case series of 4 patients)	10-15	3 female, 1 male	Not fully specified	Three orbital lesions in female patients and one neck lesion in a male patient	2/4 had elevated serum IgG4. Dense plasma cell infiltrate, eosinophils, perivascular fibrosis, 60-110 IgG4 positive cells/HPF, IgG4/IgG = 0.5-0.9; no storiform fibrosis or obliterative phlebitis	Treated with steroids (2), rituximab (1), and surgery (1); all improved
Tille et al., 2019^8^	16	Female	Unilateral (left)	Left orbit: optic nerve/perineural tissue, extraocular muscles, periocular soft tissue, lacrimal gland; later systemic intestinal/colonic involvement	Elevated serum IgG4. Positive antinuclear antibody (ANA). Colitis: biopsy with >10 IgG4 positive cells/HPF	Orbital disease responded to corticosteroids. Colitis developed during steroid taper and improved after methotrexate therapy
Karim et al., 2016^12^ (Systematic review of 25 patients)	Median age: 13	Female predominance (64%)	Not uniformly reported	Orbital involvement in 44%; pancreatitis 12%; cholangitis 8%; pulmonary disease 8%; systemic involvement in 40%	Serum IgG4 was elevated in 16/23 of patients (70%); nearly all cases were histologically supported	Prednisone was first-line therapy in 23/25 cases, with a rapid response observed in 19/23. Steroid-sparing therapy was required in 11/25 treated cases. In refractory disease (n = 6), successful treatment was achieved with rituximab (n = 4), adalimumab (n = 1), and cyclophosphamide plus methylprednisolone (n = 1).

Several clinicopathologic distinctions have been described between pediatric and adult IgG4-related disease. Pediatric IgG4-related disease does not appear to demonstrate the clear male predominance commonly reported in adult cohorts.^10^ In addition, surface organ involvement, particularly ophthalmic manifestations such as lacrimal gland and orbital disease, seems to be more frequently reported in children than in adults.^10^ Organ involvement in pediatric cases also tends to follow a more indolent course and may present unilaterally.^10^ Notably, because pediatric IgG4-related disease more commonly involves the orbital region, normal serum IgG4 levels are reported in a substantial proportion of children, particularly those with orbital disease, with rates of 58% in pediatric IgG4-related ophthalmic disease compared with 33% in pediatric IgG4-related disease overall.^9^ Histopathologically, both pediatric and adult IgG4-related disease share the same characteristic features, including dense lymphoplasmacytic infiltrates, increased numbers of IgG4-positive plasma cells, an elevated IgG4/IgG ratio, storiform fibrosis, and obliterative phlebitis. Based on the currently available literature, there are no well-established histopathologic differences between pediatric and adult IgG4-related disease, although the rarity of pediatric cases limits definitive comparisons.^10^^,^^15^

In summary, ophthalmologists should consider IgG4-related disease in the differential diagnosis of pediatric orbital proptosis, even in very young children, particularly when the lacrimal gland is involved.^16^ The differential diagnosis should also include idiopathic orbital inflammatory syndrome, lymphoproliferative disorders, tuberculosis, sarcoidosis, malignancies such as rhabdomyosarcoma, infections, xanthogranulomas, and Erdheim-Chester disease. In children, IgG4-related orbital disease is typically unilateral, with orbital soft tissue and extraocular muscles being most commonly affected.^5^ Nearly all reported pediatric cases were confirmed histologically, underscoring the importance of biopsy and IHC in establishing the diagnosis.

As previously recognized, corticosteroids remain the cornerstone of treatment, with excellent reported response rates (89–100%),^17^ though Taranto et al., after reviewing 105 cases of pediatric IgG4 disease, emphasized the high relapse rate of disease after glucocorticoids and advocated for the early introduction of steroid-sparing immunosuppressants to avoid relapse.^18^ In refractory cases, DMARDs and biologic agents such as rituximab or adalimumab may be considered, with rituximab showing the highest success rate (93%).^4^^,^^19^ Comprehensive systemic evaluation particularly in bilateral cases is essential to exclude multi-organ involvement, including imaging of the head, neck, chest, abdomen, and pelvis, which are more frequently associated with systemic disease.^20^

## CRediT authorship contribution statement

**Hassan Asadigandomani:** Writing – review & editing, Writing – original draft, Methodology, Data curation. **Afsaneh Malekpour:** Writing – original draft, Methodology, Data curation. **Mohammad Taher Rajabi:** Methodology, Data curation, Conceptualization. **Amirhossein Aghajani:** Methodology, Data curation, Conceptualization. **Fahimeh Asadi Amoli:** Investigation, Data curation. **Seyed Mohsen Rafizadeh:** Writing – review & editing, Visualization, Validation, Supervision, Project administration, Methodology, Investigation, Formal analysis, Data curation, Conceptualization.

## Ethics approval and consent to participate

The study was approved by the local Ethics Committee of Tehran University of Medical Sciences in accordance with the principles of the Declaration of Helsinki. Written informed consent was obtained from all parents or legal guardians.

## Consent for publication

Written informed consent for publication of deidentified clinical details and accompanying images was obtained from the patients’ parents or legal guardians.

## Availability of data and materials

All data supporting the findings of this study are included within the article.

## Funding

There were no specific funding sources for this study.

## Declaration of competing interest

The authors declare that they have no known competing financial interests or personal relationships that could have appeared to influence the work reported in this paper.
